# Activation of the Innate Immune Response against DENV in Normal Non-Transformed Human Fibroblasts

**DOI:** 10.1371/journal.pntd.0001420

**Published:** 2011-12-20

**Authors:** José Bustos-Arriaga, Jazmín García-Machorro, Moisés León-Juárez, Julio García-Cordero, Leopoldo Santos-Argumedo, Leopoldo Flores-Romo, A. René Méndez-Cruz, Francisco J. Juárez-Delgado, Leticia Cedillo-Barrón

**Affiliations:** 1 Departamento de Biomedicina Molecular Centro de Investigación y de Estudios Avanzados, México Distrito Federal, Mexico; 2 Departamento de Biología Celular Centro de Investigación y de Estudios Avanzados, México Distrito Federal, Mexico; 3 Laboratorio de Inmunología UMF de la FES Iztacala Universidad Autónoma de México, Tlalnepantla Estado de México, Mexico; 4 Departamento de Cirugia, Hospital Santa Maria Ticomán, México Distrito Federal, Mexico; Universidade de São Paulo, Brazil

## Abstract

**Background:**

When mosquitoes infected with DENV are feeding, the proboscis must traverse the epidermis several times (“probing”) before reaching a blood vessel in the dermis. During this process, the salivary glands release the virus, which is likely to interact first with cells of the various epidermal and dermal layers, cells which could be physiologically relevant to DENV infection and replication in humans. However, important questions are whether more abundant non-hematopoietic cells such as fibroblasts become infected, and whether they play any role in antiviral innate immunity in the very early stages of infection, or even if they might be used by DENV as primary replication cells.

**Methodology/Principal Findings:**

Fibroblasts freshly released from healthy skin and infected 12 hours after their isolation show a positive signal for DENV. In addition, when primary skin fibroblast cultures were established and subsequently infected, we showed DENV-2 antigen-positive intracellular signal at 24 hours and 48 hours post-infection. Moreover, the fibroblasts showed productive infection in a conventional plaque assay. The skin fibroblasts infected with DENV-2 underwent potent signaling through both TLR3 and RIG- 1, but not Mda5, triggering up-regulation of IFNβ, TNFα, defensin 5 (HB5) and β defensin 2 (HβD2). In addition, DENV infected fibroblasts showed increased nuclear translocation of interferon (IFN) regulatory factor 3 (IRF3), but not interferon regulatory factor 7 (IRF7), when compared with mock-infected fibroblasts.

**Conclusions/Significance:**

In this work, we demonstrated the high susceptibility to DENV infection by primary fibroblasts from normal human skin, both *in situ* and *in vitro*. Our results suggest that these cells may contribute to the pro-inflammatory and anti-viral microenvironment in the early stages of interaction with DENV-2. Furthermore, the data suggest that fibroblast may also be used as a primary site of DENV replication and provide viral particles that may contribute to subsequent viral dissemination.

## Author Summary

In this work, we demonstrate that that both human whole skin and freshly isolated skin fibroblasts are productively infected with Dengue virus (DENV). In addition, primary skin fibroblast cultures were established and subsequently infected with DENV-2; we showed in these cells the presence of the viral antigen NS3, and we found productive viral infection by a conventional plaque assay. Of note, the infectivity rate was almost the same in all the primary cultures analyzed from different donors. The skin fibroblasts infected with DENV-2 underwent signaling through both TLR3 and RIG-1, but not Mda5, triggering up-regulation of IFNβ, TNFα, defensin 5 (HB5) and β defensin 2 (HβD2). In addition, DENV infected fibroblasts showed increased nuclear translocation of interferon (IFN) regulatory factor 3 (IRF3), but not interferon regulatory factor 7 IRF7, when compared with mock-infected fibroblasts. Our data suggest that fibroblasts might even participate producing mediators involved in innate immunity that activate and contribute to the orchestration of the local innate responses. This work is the first evaluating primary skin fibroblast cultures obtained from different humans, assessing both their susceptibility to DENV infection as well as their ability to produce molecules crucial for innate immunity.

## Introduction

Dengue virus (DENV) has become one of the most important arthropod-borne viral infections of humans, with approximately 100 million cases per year. The etiological agent is a positive-sense, single-stranded RNA virus that belongs to the *Flaviviridae* family, of which there are four antigenically related serotypes (DENV-1, DENV-2, DENV-3 and DENV-4) [1], [2].

Cumulative data have demonstrated that both the innate and adaptive immune responses participate in the control and pathogenesis of Dengue disease, which has a wide spectrum of clinical forms ranging from weak Dengue Fever (DF) to severe dengue disease, such as dengue shock syndrome and hemorrhagic Dengue (SSD/DHF). A rapid initiation of the innate host defense may be the critical limiting step in the infection because the Dengue virus must overcome all barriers mediated by innate immunity before arriving at the regional lymphoid tissues [3].

The symptoms generally appear 4–7 days afterward; at this point, when the adaptive immune response may be ongoing, the patients usually present for medical care. As a result, much work has been devoted to understanding the adaptive immune response to DENV, but not enough information has been raised about the very early steps of interaction with the host [4]–[6].

One of the most intriguing questions about dengue is the identity of the non-hematopoietic cells that may play a crucial role in the innate antiviral immune response to DENV in the early stages of infection. Given the fact that mosquitoes inoculate DENV into human skin while they are feeding, the potential target cells for dengue infection should be localized in the dermis and epidermis, which constitute the first level of defense [7].

The more abundant cells present in the inoculation site are keratinocytes and fibroblasts. These cells could act either as a limiting step or like a jumpstart of the replication cycle, depending on inoculation multiplicity, and intrinsic host variability. Early studies infecting human forearm dermal fibroblasts have suggested the participation of skin fibroblasts in the immune response against DENV [8].

Although dendritic cells and monocytes in the skin have been suggested as important targets of DENV infection, the number of these cells is considerable low compared with fibroblasts [9], [10].

The skin is not only a physical protective barrier; it also participates in the rapid initiation of innate host defenses that might represent a limiting step to DENV infection. In the skin, both the infiltrating cells (such as macrophages, neutrophils, dendritic cells and lymphocytes), and the resident cells, such as the keratinocytes and fibroblasts that are abundantly localized in the epithelia, participate in the production of various types of cytokines, establishing a pro-inflammatory microenvironment with antimicrobial activity against arthropod borne pathogens such as enveloped viruses [11]. Although some of these elements have been exhaustively reviewed by Nielsen *et al*. [12], little is known about the events that occur in the skin in the very early stages after mosquito feeding.

It is conceivable that DENV has evolved mechanisms to evade the early innate host responses, which are initiated by pattern recognition receptors (PRRs). Among the PRRs the family of membrane–bound Toll-like receptors (TLRs) [13] and the RIG-I like receptors (RLR); Retinoic acid-inducible gene (RIG-I) and the melanoma differentiation-associated gene 5 (Mda5) [14]. PRRs triggering lead to a signaling cascade that converges on the activation of latent transcription factors, such as interferon response factor 3 (IRF3), interferon response factor 7 (IRF7) and nuclear factor kB (NF-kB) activating transcription factor 2 (ATF-2)/c-Jun. The transcription factors undergo subsequent nuclear translocation and bind to the type I interferon's (IFNα/β) gene promoters. Then activated homo- and hetero-dimers of IRF3 and IRF7 bind to the IFN-stimulated response elements (ISRE) located in the promoter regions of various ISGs, including ISG54, ISG56 and ISG15, thereby enabling direct IFN-independent activation of these ISGs. The synthesis of type 1 IFNs, in turn leads to the development of an antiviral state in the surrounding cells and to the activation and modulation of the adaptive immune system [15]–[17].

Cumulative data have shown that DENV is sensed by both TLR3 and TLR7. At this respect Warke et al., demonstrated that plasmacitoid dendritic cells (pDCs) constitutively express both TLR7 and through IRF7 lead to IFNα/β production in response to DENV. By contrast, HUVEC cells or U937 cells that have internalized DENV produce IL-8 and IFNα/β after viral recognition through TLR3 [18]–[20].

Cytoplasmic molecules, such as RIG-I (which senses short double-stranded blunt-end 5′-triphosphate RNA) and MDA5 (which recognizes long dsRNA), have been shown to play an important role in sensing different flavivirus, including DENV. Recent experiments using single and double RIG-I/MDA5 knockout, fibroblast cell lines have shown that DENV triggers the interaction of both molecules with the IPS-1 molecule to activate IRF3, IkB kinase and phosphatidylinositol-3 kinase (PI3K) [21], [22]. In addition, Conceicaoi et al., showed that TLR3, TLR8, RIG-I and MDA5 mRNA are up-regulated, along with the type I interferon IFNβ and pro-inflammatory cytokines, in HepG2 cells infected with DENV [23].

The IFN response is one of the early mechanisms of host defense that contributes significantly to innate immunity. The IFN system includes cells that synthesize IFN in response to viral infection. The induction of IFNα or β is one of the early events that follows viral infection, and it is widely accepted as the most immediate and important antiviral host response to many viral infections [24].

Indeed, mice deficient in IFNα/β and IFNγ receptors are more susceptible to mortality following intraperitoneal inoculation of mouse-adapted DENV [25], [26]. A strong IFNα-mediated inhibitory effect on DENV replication was also observed when different cell types were treated with IFNβ prior to virus exposure [27].

Recently, antimicrobial peptides have been considered as a key element in the defense mechanisms of the skin. Cumulative data had shown that defensins modulate the immune response against both enveloped and non-enveloped virus, by inducing cytokine and chemokine production and inflammatory and immune cell activation [28], [29]. In this study, we demonstrated that skin fibroblasts freshly released from healthy human skin and subsequently infected with DENV showed positive viral antigen at 48 h post-infection.

Considering that fibroblasts are one of the most abundant dermal cells in the host during early contacts with this virus, primary skin fibroblast cultures were established to address the contribution of these cells in innate immunity. Our findings revealed that fibroblasts sense the DENV through TLR3 and RIGI which then signal to produce IFNβ, TNF, and β defensins. We believe these are previously unrecognized features of importance in the immunobiology of DENV infection.

## Materials and Methods

### Cells and DENV-2 virus

The DENV-2 clinical isolate that we used has been described previously [5]. Mosquito C6/36 cells derived from *Aedes albopictus* were grown in MEM supplemented with 10% fetal bovine Serum (FBS) (Gibco Carlsbad, CA) at 34°C. Baby hamster kidney (BHK-21) cells were cultured at 37°C in the presence of 5% CO_2_ in MEM supplemented with 10% FBS, 1 IU penicillin/mL, 1 µg/mL streptomycin and 2.4 ng/mL of amphotericin B at final pH of 8. The virus stock was prepared by infecting a C6/36 cell monolayer in 75 cm^2^ tissue culture flasks at 75%–85% confluence. When the infected monolayer showed cytopathic effects, the cells and supernatant were homogenized and diluted in a 40% polyethylene glycol solution in 2 M NaCl (Sigma-Aldrich St. Louis, MO) and incubated at 4°C overnight. The suspension was centrifuged at 6000 rpm for 1 h. The virus was resuspended in 1/15 of the total volume with a glycine buffer (Tris 50 mM, Glycine 200 mM, NaCl 100 mM and EDTA 1 mM) and 1/30 of the total volume of FBS. The virus was homogenized, aliquoted and frozen at −70°C until use.

The virus was titrated by the standard plaque-forming assay technique using BHK-21 cells as described elsewhere. Briefly, ten-fold serial dilutions of virus stock in Hank's salt solution (Gibco Carlsbad, CA) were used to infect monolayers of BHK-21 cells in 24-well plates. After incubation at 37°C for 1 h, the infected cells were overlaid with MEM Eagle modified medium (Gibco Carlsbad, CA) with 3% carboxymethyl cellulose (Sigma-Aldrich St. Louis, MO). After 5 days, the resulting plaques were stained with naphthol blue-black solution to quantify the plaque forming units (PFUs).

### Immunohistology

Freshly prepared, non-cadaveric, healthy human skin was obtained at the Surgery Department of the Hospital General de Ticoman (the protocol was approved by the hospital ethical committee) from patients submitted to general surgery; the tissue was obtained from the wound area during the surgical procedure.

For the histology, the biopsies were washed extensively with Dulbecco containing penicillin (200 mg/mL) and streptomycin (200 U/ml) as described by Limon-Flores et al [5]. The tissue was then cut into pieces of approximately 1.0 cm^2^, the underlying fat was carefully removed and each piece was placed (epidermis side up) into individual wells in a 12-well culture plate (Costar corning, NY, USA). Skin explants were gently inoculated with 1×10^6^ PFU of the virus in a total volume of 30 µL using a 30-gauge insulin syringe needle (HSW, Tuttlingen, Germany) in the upper side of the epidermis, causing penetration into the dermis without passing through the explants. Control explants were inoculated with either UV-inactivated DENV or with PBS for the manipulation control.

The inoculated explants were incubated in a 24-well plate for 24 and 48 h with DMEM containing 15% FBS and supplemented with antibiotic, antimycotic, non-essential amino acids and L-glutamine (Gibco Carlsbad, CA) in an incubator with a humid atmosphere and 5% CO_2_ at 37°C. At the end of the incubation, the skin was mounted in tissue freezing medium (Jung 0201 08926). The histological sections were obtained as described elsewhere [5].

### Tissue immunofluorescence

The explants where cut into 10 µm frozen sections. The tissue sections were fixed with 4% paraformaldehyde (Sigma-Aldrich St. Louis, MO) for 1 h. After 4 washes with PBA, the sections were blocked with goat serum at 10% in PBA for 1 h. The primary and secondary antibodies were incubated for 1 h. After 5 washes between each antibody, the samples were mounted and analyzed using a confocal microscope (Olympus FXM).

### Cell suspension from fresh skin and immunofluorescence

The fat tissue was removed carefully from the normal skin explants with a scalpel. The tissue was then cut into pieces of approximately 1 mm^2^ and washed exhaustively with DPBS (Sigma-Aldrich St. Louis, MO) supplemented with an antibiotic solution (3 IU/mL of penicillin, 3 µg/mL of streptomycin and 7.2 ng/mL of amphotericin B). Afterward, the tissue was incubated with a collagenase/dispase cocktail at 37°C in agitation. The cells in suspension were collected at 12 h and 24 h post-treatment. All cells were split into two 24-well plates over a slide treated with poly L-Lysine. They were then incubated for 12 h at 37°C in a 5% CO_2_ atmosphere. The cells were infected with 1×10^6^ PFU of the virus and the immunofluorescence was performed as described below. The cells were double-stained for fibroblasts (Sigma-Aldrich St. Louis, MO) and for the dengue envelope protein (Chemicon Millipore Billerica, MA). Mouse anti-human IgM-rhodamine (Jackson Pennsylvania, Phi), and goat anti mouse IgG2a-FITC (Southern Biotech Birmingham, AL) were used as the secondary antibodies.

### Establishment of primary cultures of fibroblasts from human skin explants

The explants where cut into pieces of approximately 2 mm^2^, and the tissue were incubated for 4.5 hours in a cocktail of collagenase and dispase (0.5 U/mL and 4 U/mL, respectively). After this treatment, the epidermis was mechanically removed from the dermis and incubated separately in a 0.1% trypsin solution (Sigma-Aldrich St. Louis, MO) for 30 minutes. The cell suspension and tissue fragments were then resuspended in DMEM medium (Gibco Carlsbad, CA) containing 20% fetal calf serum (FCS) and 10 ng/mL epidermal growth factor (Gibco Carlsbad, CA) in a 25-cm^2^ flask. After incubation for 5 to 10 days, when adherent cells covered the plastic flask, the tissue was removed and the cells were maintained in DMEM medium (Gibco Carlsbad, CA) containing 15% FCS and 10 ng/mL epidermal growth factor (Gibco Carlsbad, CA). The culture was passaged until confluence was reached. In addition, surface fibroblast protein marker expression was checked for each established primary culture. The cultures were used between the passages 2–10.

### Flow cytometry staining

Infection of the skin fibroblasts was analyzed by flow cytometry. Briefly, 2×10^5^ primary human skin fibroblasts were infected with 5 PFU/cell of DENV-2. Six hours before harvesting, the cells were treated with brefeldin A (Sigma-Aldrich St. Louis, MO) to interrupt the vesicular traffic. After 3 washes, the cells were fixed and treated with a permeabilizing solution (Becton Dickinson, Franklin Lakes, N.J) for 45 minutes. The cells were analyzed at 6, 12, 24 and 48 hours post-infection. All the cells were treated for 30 minutes with a non-related immunoglobulin solution (10% goat serum in PBS) to block the Fc receptors. The primary antibodies were diluted in blocking solution (PBS-10% Goat serum) at the corresponding dilutions: E protein (Chemicon Millipore Billerica, MA), IFNβ (Santa Cruz CA, USA), APC-TNFα (Serotec Kidlington UK), human β defensin 2 (Santa Cruz CA, USA), human defensin 5 (Santa Cruz, CA, USA), TLR3 (ebiosciences San Diego, CA), RIG-I (Santa Cruz CA, USA), Mda5 (Santa Cruz CA, USA) and fibroblasts surface protein (Sigma-Aldrich St. Louis, MO). The non-conjugated antibodies were marked with the corresponding secondary antibodies: anti-mouse IgG H+L (Caltag Invitrogen Carlsbad CA), sheep IgG H+L (Invitrogen, Carlsbad CA), anti-rabbit IgG H+L (Invitrogen, Carlsbad CA), and anti- goat IgG H+L Zymed Invitrogen Carlsbad CA). For flow cytometry, the cells were resuspended in 0.01% EDTA in PBS. Data were collected using a FACS Calibur flow cytometer (Becton Dickinson, Franklin Lakes, N.J) and analyzed using FlowJo software (Tree Star, Inc.). All the results were statistically analyzed using *t* student test with graph pad Prism 5 software (CA, USA).

### RNA extraction and RT-PCR

Total RNA was extracted at the indicated times from mock- or DENV-infected cells Poly I:C (Amershan USA) transfected cells (as positive control) using TRIZOL reagent (Gibco Carlsbad, CA), according to the manufacturer's instructions. The RNA concentration was measured by spectrophotometry, and the quality of the purified RNA was analyzed. Total RNA (2 µg) was used for DNase (Invitrogen, Carlsbad CA) digestion, and RT for the synthesis of the first strand of cDNA was performed using SUPERSCRIPT (Invitrogen, Carlsbad CA) in a final volume of 30 µl. Five percent of the first-strand reaction was used for the PCR analysis. The PCR amplification was performed using specific primers for RIG-I (5′ GCATATTGACTGGACGTGGCA-3′ and 5′ CAGTCATGGCTGCAGTCC TGTC-3′), TLR3 (5′-CCCTTGCCTCACTCCCC-3′ and 5′-CCTCTCCATTCCTGG CCT-3′), TLR7 (5′-CCTCAGCCACAACCAACTG-3′ and 5′-TTGTGTGCTCCTG GCCCC-3′) and GAPDH (5′-GACCCCTTCATTGACCTCAAC-3′ and 5′-GTCCATGCCCATCA CTGCCAC-3′). The PCR products were separated by 1% agarose gel electrophoresis. The assays were performed at least three times from different RNA preparations.

### Immunofluorescence of cell cultures

The cells were seeded on glass cover slips (6×10^4^) (Bellco NJ USA). After 24 h, the culture medium was removed and monolayers were infected with DENV-2 active or UV-inactivated virus both at 5 PFU per cell; they were then incubated at 37°C and analyzed by immunofluorescence at different times. Briefly, the cells were fixed with 4% *para*-formaldehyde (Sigma-Aldrich St. Louis, MO) in PBS for 20 minutes at room temperature; the cells were then permeabilized with 0.1% Triton-×100 in PBS and blocked with 10% normal goat serum. The cell monolayer was treated for 60 minutes with primary antibody: E protein (Chemicon Millipore Billerica, MA), NS3 [30], NFkB p50 (Novus Biologicals), IRF3 (Santa Cruz CA, USA), IRF3 ser396 (Cell Signaling 4D46 4947S), IRF7 (Santa Cruz CA, USA) and fibroblasts surface protein (Sigma-Aldrich St. Louis, MO) followed by fluorochome-conjugated secondary antibody mouse IgG H+L (Caltag Invitrogen Carlsbad CA), rabbit (Invitrogen, Carlsbad CA), and goat (Zymed Invitrogen Carlsbad CA). An irrelevant isotype antibody that matched the monoclonal antibody was used as a negative control.

Finally the nucleus was labeled with DAPI (1 µg/mL) in PBS for 10 minutes and the slides were mounted with Vectashield (Vector). The images were captured using two different confocal microscopes (Leica SP2 and OLYMPUS FVX).

### ELISA assay to detect IFNβ in primary fibroblast cultures

Primary skin fibroblasts were seeded and infected at 5 MOI as described previously. The supernatants were harvested at 12 h post-infection for IFNβ (Interferon source, NJ USA) and at 24 h for TNFα (Biolegend NJ USA). The cytokine levels were measured according to the manufacturer's instructions. Absorbance at 450 nm was measured using the ELISA reading equipment (Sunrise Tecan, Salzburg, Austria).

### Terminal deoxynucleotidyltransferase-mediated dUTP nick-end labeling (TUNEL) assay

Apoptosis-induced DNA strand breaks were end-labeled with dUTP terminal deoxynucleotidyltransferase using a commercial kit, according to the manufacturer's instructions (*In Situ* Cell Death Detection kit, TMR red; Roche Indianapolis, IN) Briefly, the cells were fixed with *para*-formaldehyde [2% in phosphate-buffered saline (pH 7.4)] for 60 min at room temperature and permeabilized in 0.1% Triton X-100–0.1% sodium citrate for 2 min in an ice bath. The TUNEL reaction was performed using TMR red dUTP at 37°C for 60 min, and the labeling was analyzed by fluorescent microscopy. TUNEL assays with infected and mock-infected cells with different DENV 2 proteins were performed at 24 and 48 h post-infection. As a positive control, human primary cultured skin fibroblasts were exposed to 10 µg/mL DNase for 10 min at room temperature.

## Results

### DENV is present in human skin explants inoculated with DENV-2

**Figure 1:**
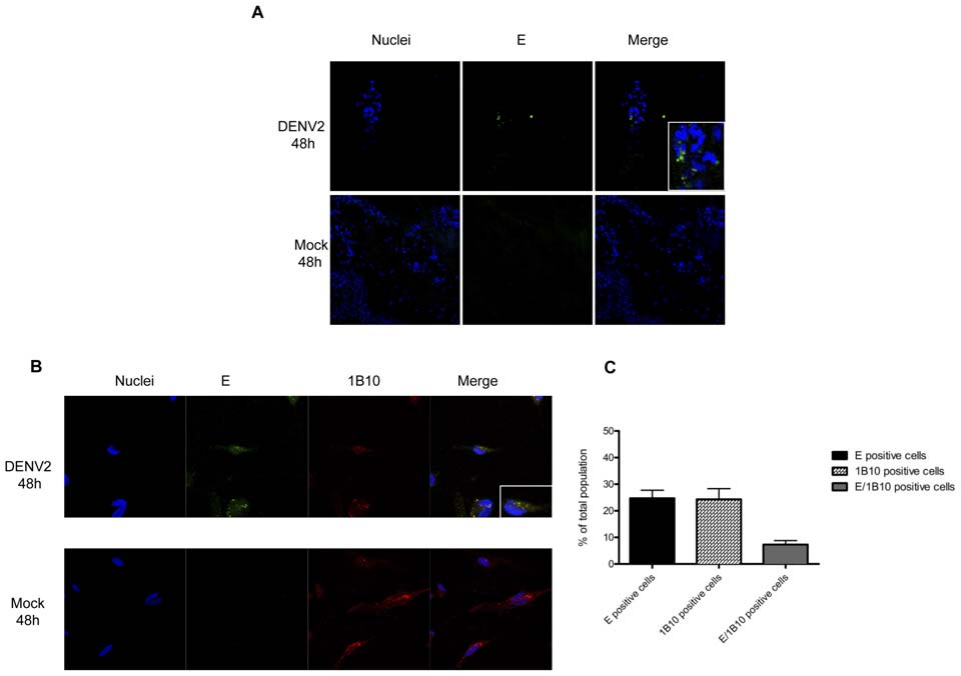
Infection of normal skin cells by the Dengue virus. A) Skin explants were inoculated (with 1×10^6^ PFU in a total volume of 30 µL) with a 30-gauge insulin syringe needle from the epidermis side. After 48 h of incubation, sections of 10 µm were stained for the presence of E glycoprotein (green) in mock-infected (Mock) and, DENV-2 infected skin primary fibroblasts. B) Human skin cells were obtained from fresh skin explants. The cells in the suspension were collected at 24 h after the enzymatic treatment. Afterwards, the suspension was left in complete culture medium for 12 h to allow for the recovery of receptors, the cells were then infected (DENV), or mock infected with 1×10^6^ PFU of the DENV. After 48 h double staining was performed for the presence of envelope protein (green fluorescence) and Fibroblast protein (red fluorescence). The nucleus was contrasted with DAPI (blue fluorescence) C) Quantitation of DENV infected fibroblasts cells was determined by microscopic analysis. Two hundred cells from each experiment were counted on three different fields of cells suspension. Four different cells suspension were analyzed.

To investigate whether healthy human fibroblasts from the skin are infected *in situ* with DENV-2, we employed a model previously reported by our group using non-cadaveric fresh skin explants [5]. The tissue samples were either infected with 1×10^6^ DENV-2 or mock-infected, and 24 hours later analyzed by immunofluorescence. The DENV-2-infected tissue showed cells with viral antigen (E protein in green) in the dermis area; these cells were likely dermal fibroblasts due to their morphology and location. By contrast, no expression of E protein was detected in skin explants incubated with the UV-inactivated virus (mock 48 h), as shown in Figure 1A.

To verify this result, healthy human skin was processed as described in Materials and Methods to obtain fresh skin cell suspensions. These cells were incubated for 12 hours before infection to allow the re-expression of cell receptors that might have been lost during the colagenase/dispase treatment. The Skin cell suspensions were then infected with 1×10^6^ PFUs and analyzed 48 h post infection. Skin fibroblast infection was assessed by immune detection of E protein (green); the monoclonal antibody 1B10, which is specific for a fibroblast surface protein, was used to identify fibroblasts (red). Figure 1B clearly shows that the fibroblasts from healthy skin are permissive to DENV-2 infection; in contrast to mock-infected dermal suspensions, where only the fibroblast marker (red) was observed. Of note, 7% of infected cell suspension that were positive for fibroblast marker, also showed a positive labeling for DENV antigen E, Thus corroborating the above results in which we also detected viral antigens in the *in situ* zone where fibroblasts are located Figure 1C. These data warranted further investigation regarding fibroblasts, as these may be one of the cell types that first support dengue virus and other flavivirus infections *in vivo* after the bite of flavivirus-infected mosquitoes.

### Establishment of primary skin fibroblasts cultures

It is well known that established immortalized cell lines may have multiple mutations that can affect the permissiveness to viral infections [31], [32]. In addition, fibroblasts are one of the more abundant cell types in the skin where the mosquito introduces the DENV. Thus, primary skin fibroblast cultures should be an appropriate in vitro model to provide insights about the role of if these cells in skin.

We established 10 primary cultures of skin fibroblasts from 10 different donors who were free from any known infectious disease and especially had no dermatological disorders. Skin biopsies were obtained from these donors who consented to participate in this study. The characteristics of the surgery, age, and the permissiveness of DENV infection as well as general information about the donors are included in the Figure S1A.

Each cell culture was first characterized by flow cytometry, assessing the expression of fibroblasts cell surface protein marker with the mAb 1B10, (Figure S1B). This finding confirmed that the cultures of established skin fibroblasts consisted of a homogeneous population of cells expressing the protein marker recognized by 1B10 antibody. HMEC-1 cells (endothelial cells) and isotype control staining were used as the corresponding controls. All the experiments were carried out on fibroblasts with less than 10 passages, because according with our data after more than 15 passages the cells become highly permissive to DENV infection, indicating changes regarding the original cells. Our data show that infection of primary skin fibroblasts from passages 2–14 did not exhibit overt changes in the percentage of infection (data no shown).

**Figure 2:**
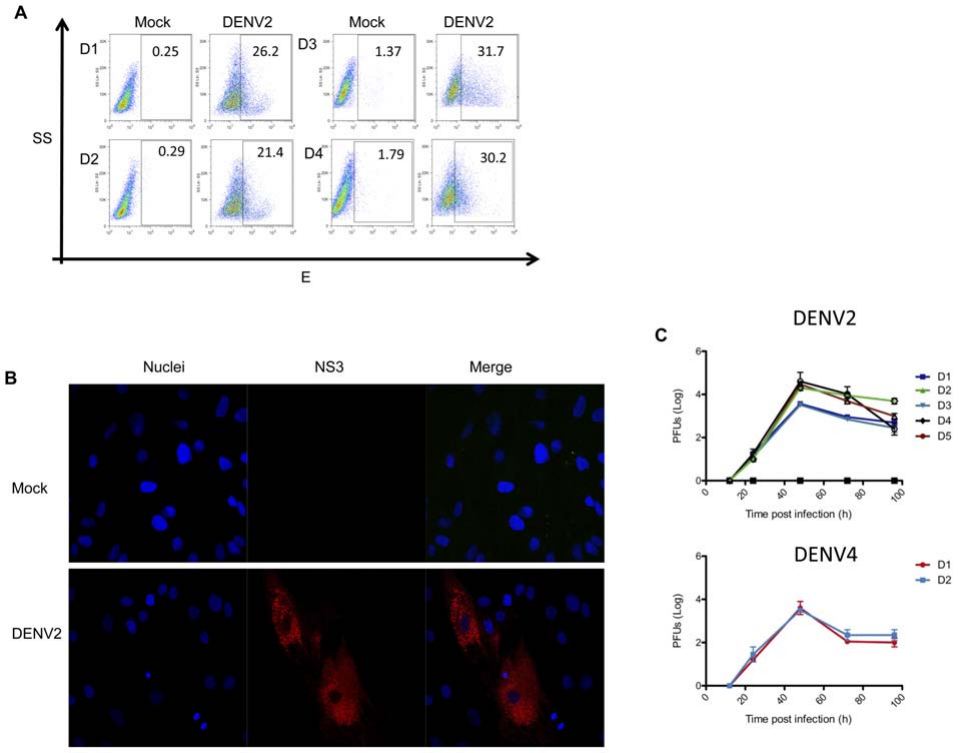
Skin fibroblasts from different donors are permissive to DENV-2 infection and replication. A) FACS analysis to assess the permissiveness of four representative primary skin fibroblasts from different donors (D1–4) infected with DENV at 5 MOIs. E protein expression was analyzed at 48 h post-infection using a specific monoclonal antibody. The percentage of positive cells is given for each condition, for mock-infected (Mock) and, DENV-2 infected, primary skin fibroblasts. B) Primary skin fibroblasts were infected and mock-infected with DENV-2 or DENV-4 (H241) at MOI 5, and immunofluorescence staining was performed to detect NS3 at 48 h post-infection (red). The nuclei were stained with DAPI (blue). C) Skin fibroblasts from four healthy donors (D1–4) were infected with DENV-2 and DENV-4 at MOI 5 and analyzed at 12, 24, 48, 72 and 96 h post-infection for DENV virus yields by titrating infectious viral progeny on permissive BHK cells. Each line represents a different donor.

Once the primary cultures were established and well characterized, they were infected with DENV-2 at MOI of 5. The cells were then harvested and the expression of E protein was analyzed by flow cytometry at different post-infection times. To address whether the origin of the subjects skin biopsy may have influenced the susceptibility of the fibroblasts to infection with DENV, ten different primary cultures obtained from ten different subjects were infected or mock-infected, and the data are shown in the Figure S1C. Figure 2A shows the FACS results of 4 representative infected primary cultures of dermal fibroblasts expressing the DENV viral E protein with data from 3 independent experiments. The graphics corresponding to the rest of the cultures are shown in the Figure S1C; according to the number of viral E protein antigen-positive cells, the range of infection fluctuates from 21.4% to 31.7% regardless of the donor source. No E protein expression was detected in the mock-infected cells (Figure 2A). To further investigate whether the DENV replicated in the primary fibroblasts, different cultures were infected or mock-infected and then analyzed by immunofluorescence using a mouse monoclonal antibody to the NS3 protein [30]. Figure 2B shows that only the fibroblasts inoculated with active DENV-2 showed positive staining for NS3. By contrast, no staining was observed in the mock-infected cells or the isotype control cells. Furthermore, a plaque assay was performed to demonstrate that the fibroblasts supported active DENV viral replication.

Previous works have shown variation in the susceptibility to infection among cell types, using different DENV-2 strains. While some cells are susceptible to infection by all DENV2 strains assayed, human foreskin fibroblasts showed differences in the susceptibility to the prototype viral Thai isolates and Nicaraguan strains, depending on the passage [33]. In order to address this point we additionally infected four different established skin fibroblasts with a reference type virus (DEN4 H241). There were no significant differences in the viral titers obtained from different fibroblast cultures, and each line showed similar viral replication kinetics regardless of the donor (Figure 2C).

The results presented here strongly support the notion that skin fibroblasts must be one of the primary cell types that can hold up initial dengue virus infections *in situ*, during or immediately after the bite of a DENV-2-infected mosquito.

### PRR(s) are involved in the fibroblasts detection of DENV

Previous reports have demonstrated that the TLR3 and TLR7 molecules are involved in flavivirus recognition. These molecules are predominantly observed in the intracellular compartments. Meanwhile, RIG-I and MDA5 are cytoplasmic sensors involved in the recognition of RNA. Furthermore, the above mentioned molecules were demonstrated to have a role in the innate immunity against DENV-2 in a mouse embryonic fibroblast model [22]. However, the variation among virus strains/serotypes or cell types may suggest distinct cellular responses. Furthermore in this study, we addressed whether human primary skin fibroblasts sense DENV through different PRRs than have been reported for different cell lineage, including MEFs.

**Figure 3:**
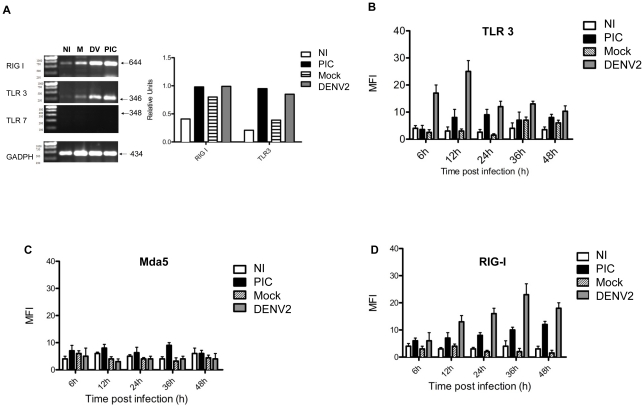
The pattern recognition receptors for viral RNA are up regulated by the dengue infection of skin fibroblasts. A) Specific amplicons corresponding to RIG-I TLR3 and TLR7 were assessed in DENV infected skin fibroblasts from different donors at 5 MOI. At 18 h post infection the cDNA was synthesized from mRNAs. Results were analyzed using the program Image J 1.410/Java 1.6.0 software and expressed in relative units for mock-infected (M), DENV-2 infected, uninfected (NI) and Poly I:C transfected primary skin fibroblasts (the control assay) (PIC). Three different donors were analyzed to evaluate, by flow cytometry, the skin fibroblasts expression level of the corresponding proteins TLR3, B) Mda5, C) and RIG-I D) for mock-infected (Mock), DENV-2 (infected at 5-MOI), uninfected (NI) and Poly I:C transfected (PIC). Bars represent standard errors for the four independent experiments from a representative donor.

Semi-quantitative RT-PCR analysis was performed in DENV-infected fibroblasts (Figure 3A). The mRNA levels of both TLR3, and RIG I showed changes at 18 hr post-infection and densitometry analysis was performed on these data. The results suggest that both TLR3 and RIG signaling are required for activating of innate response and for establishing an antiviral state in skin fibroblasts in response to DENV-2 infection. To verify the RT-PCR results, a flow cytometry assay was performed in DENV infected primary skin fibroblasts. We confirmed RIG-I up-regulation in skin fibroblasts at 12 h post-infection with a maximum at 36 h post infection, whereas there were no significant changes in RIG, TLR3 and Mda5 expression in the mock-infected cells or the non-treated cells (Figure 3D, C, D). By contrast, changes in the infected cells regarding expression of TLR3 molecule were observed as early as 6 h post-infection, with the maximum level occurring at 12 hours post-infection. Subsequently, both RIG-I and TLR3 protein levels were stabilized, and no important changes were observed in the Mda5 molecule. Our results suggest that both RIG-I and TLR3 may act in concert to detect DENV RNA in primary skin fibroblasts cultures (Figure 3D and 3B).

### Dengue infection induces IFNβ in primary human fibroblasts

**Figure 4:**
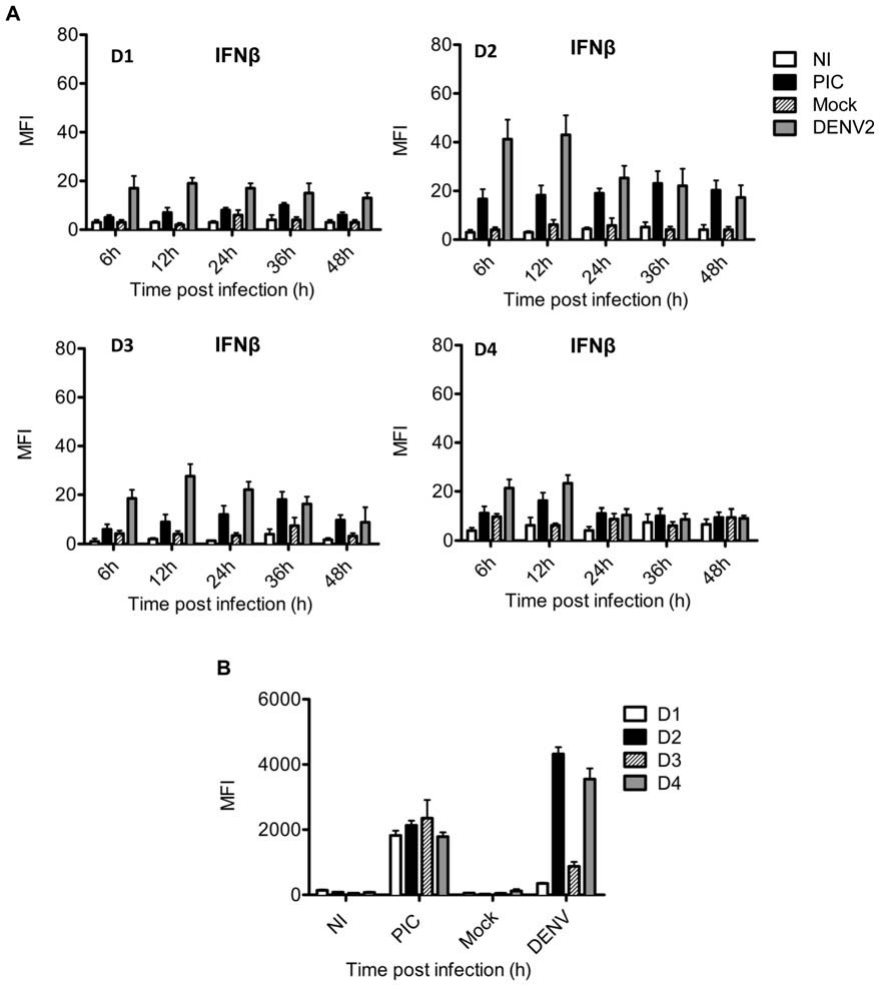
Variability in IFNβ protein production within donors after mock- or DENV-infection of primary skin fibroblasts. A) mean fluorescence intensity corresponding to the intracellular staining for IFNβ expressed by four different primary cultures obtained from different donors B) IFNβ protein levels were measured at 12 h by a specific ELISA in supernatants at 12 h from mock-infected (Mock), DENV-2 (infected 5MOI), uninfected (NI) and Poly I:C transfected (PIC) primary skin fibroblasts. Error bars indicate the standard deviations for the three independent experiments from the four representative donors.

In keeping with the other evidence presented in this article that a fresh skin fibroblast primary culture is highly permissive to active infection with DEN-2 virus, we evaluated the ability of DENV-2 to elicit a functional antiviral response. The induction of type I interferon (IFNα/β) is an early protective event that occurs within hours of viral infection and is widely accepted as one of the most immediate and important antiviral host response [34]; therefore, we investigated whether human skin fibroblasts produce IFNβ upon infection with DENV-2. A flow cytometry assay was performed, and Figure 4A shows primary skin fibroblasts from four different donors (D1, D2, D3 and D4); three independent experiments were performed with each culture and the results are presented as the mean fluorescence intensity (MFI). Figure 4A clearly shows differences among the donors. However, all primary cultures analyzed consistently showed that the infected fibroblasts expressed significant levels of IFNβ within 6–12 h of infection (Figure 4A); the expression decreased at 24 h in inverse proportion to the amount of virus. Indeed, in some of the primary cultures, the MFI of the IFNβ produced by infected fibroblasts (50 MFI) was higher than that of the IFNβ produced by poly I:C-stimulated cells (20 MFI), used as a positive control. These results contrast to the low levels observed in the uninfected and mock-infected cells.

Furthermore, we analyzed the levels of IFNβ in the culture supernatants of primary skin fibroblasts of four donors (D1–4) (Figure 4B), and we found that the cells of D2 and D4 donors produced high level of IFNβ (4800 pg/mL 3600 pg/mL) which partially corroborated the cytometry results. Although, all of the skin fibroblasts showed similar IFNβ expression kinetics, the total population was displaced in the histograms, showing a clear paracrine effect because not all the cells were infected with DENV. There were clear differences in the magnitude of the response, although the susceptibility to infection was almost the same in different fibroblast cultures.

### Dengue virus induces TNFα in primary cultures of human skin fibroblasts

**Figure 5:**
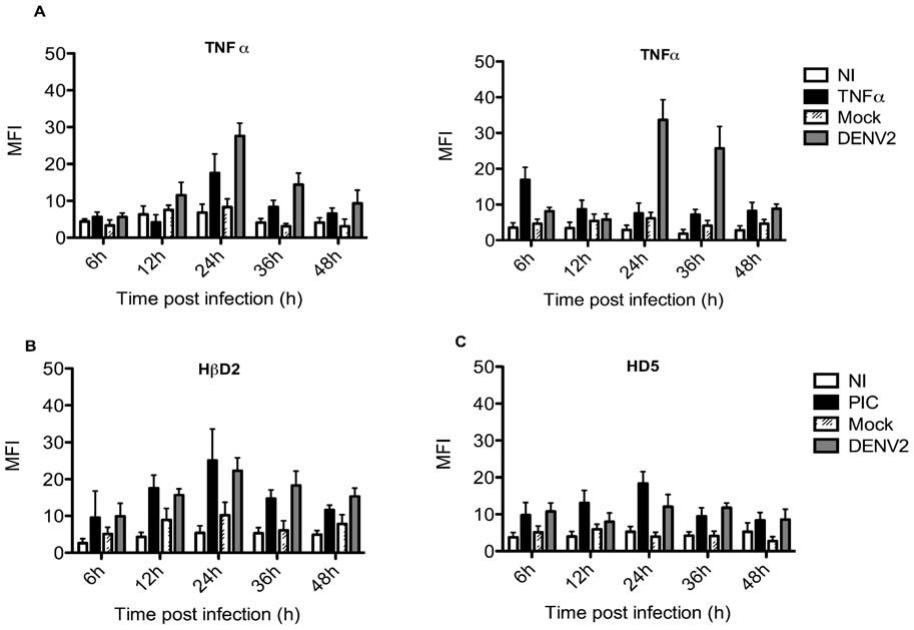
Production of TNFα, HβD2 and HD5 after mock or DENV-infection of primary skin fibroblasts. A) Mean fluorescence intensity corresponding to the intracellular staning for TNFα expressed by non-infected (NI), recombinant TNF-treated (TNFr), mock-infected (Mock) and DENV-2 infected (DENV-2) cells from two different primary cultures obtained from different donors. B) Mean fluorescence intensity corresponding to the intracellular staining for human β defensin (HβD) and **C**) Human alpha-defensin (HD5) produced by non-infected (NI), Poly I:C transfected (PIC), mock-infected (Mock) and DENV-2 infected (DENV-2) cells.

TNFα has been shown to induce inflammation and apoptosis, thereby limiting viral infection, through a wide variety of mechanisms. Indeed, TNFα can have a direct effect on viral tropism by altering the expression of the cell surface receptors used by viruses; and depletion of this cytokine using TNFα blockers might facilitate the development or reactivation of the viral infection [35]–[37]. Thus, we evaluated the expression of TNFα by the fibroblasts at various times after infection. Figure 5A shows the results of two representative primary cell cultures analyzed by flow cytometry; the results are presented as MFI. The fibroblasts expressed significant levels of TNFα 24 h post infection. In contrast, cells treated with UV-inactivated DENV-2 (mock-infected) and uninfected cells did not show any changes. Similar trends were seen in all cell lines.

### Human skin fibroblasts release defensins upon infection with DENV-2

Antimicrobial peptides have been demonstrated to be an important early element of innate immunity. Emerging studies indicated that certain defensins can block viral infections. Thus, we decided to investigate whether human primary fibroblasts were able to produce defensins during DENV infection. The production of defensins was evaluated at different times post-infection. The infected cells were able to produce HβD2 as early as 12 h post infection, reaching maximum production at 24 h (Figure 5B). DENV-2 infection also induced the production of HD5 at 24 h; however, the MFI was about half of the corresponding value for HβD2 (Figure 5C).

### Dengue infection induces early nuclear translocation of IRF3 but not IRF7 in primary human fibroblasts

The transcriptional activation of innate immunity molecules is dependent on the activation of a family of transcriptional factors from the NFkB and IRF families. Although IRF3 is ubiquitously expressed in most cell types, the expression of IRF7 is differentially regulated, depending upon both the pathogen and host cells. Because IFNβ was produced by skin fibroblasts in response to dengue infection, we decided to assess the regulatory mechanisms of this IFN induction. Our first step was to evaluate the role of the master transcriptional factors IRF3 and IRF7.

**Figure 6:**
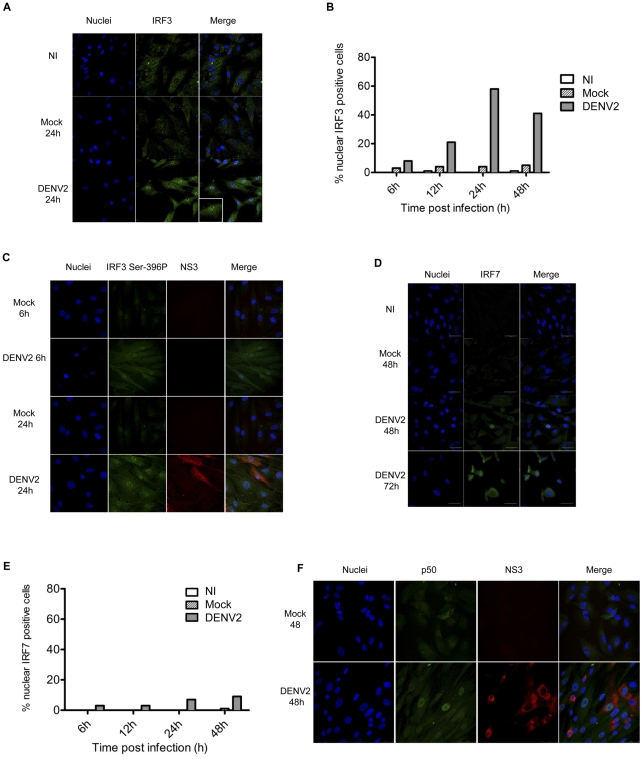
Activation of innate immunity signaling pathways in healthy skin fibroblasts infected with DENV-2. A) Localization of the transcription factor IRF3 on non-infected fibroblasts (NI), mock-infected fibroblasts (Mock) and infected fibroblasts (DENV-2) at 24 h post infection. B) Cells were fixed at different times post-infection and assayed for IRF3 localization by immunofluorescence. The cell nuclei were counterstained and the percentage of cells that microscopically showed an IRF3 increment in the nucleus area at 6, 12, 24, 36, and 48 h post infection were counted. C) Detection of activation of IRF3 in skin fibroblasts, they were stained with an antibody against active associated phosphorylation (SER 396) and double stained with an antibody against non-structural protein 3 (NS3) for mock-infected (Mock) and 5-MOI, DENV-2 infected cells. D) Localization of the transcription factor IRF7 in non-infected fibroblasts (NI), mock-infected fibroblasts (Mock) and infected fibroblasts (DENV-2) at 24 h post-infection. E) Percentage of cells that showed an IRF7 increment in the nucleus area at 6, 12, 24, 36, and 48 h post-infection. F) Localization of p50 in fibroblasts infected with DENV. Skin primary fibroblasts were infected at 5 MOI and 48 hours post-infection immunofluorescence was performed for the localization of p50 NFkB subunit (green) and NS3 (red).

For this, human skin fibroblasts were infected with 5 MOI of DENV-2. Cellular localization of IRF3 and IRF7 was analyzed by immunofluorescence at different times post-infection. Figure 6A and 6B show that fibroblasts undergo nuclear accumulation of IRF3 following infection with DENV-2. While in mock and uninfected cells, IRF3 distribution was predominantly cytoplasmic. By contrast, DENV infection of skin fibroblasts failed to induce IRF7 nuclear translocation at 24 or 48 h post-infection (Figure 6E), the location of the molecule was mainly cytoplasmic. At 72 h, when some changes were detected, the cells were already damaged and highly granular (Figure 6D).

According with the data described above IRF-3 is likely activated since 12 h post-infection. However IRF3 has to be phosphorylated to dimerise before translocating into the nucleus. To asses this, immunofluorescence was performed again with an anti-IRF3 antibody to detect active associated phosphorylation (SER 396). Nuclear IRF3 was observed mainly in the infected cells as early as 6 h post-infection increasing at 24 h post-infection, contrasting with the mock-infected or the uninfected cells where no nuclear signal was observed (Figure 6C)

### Dengue infection induces early nuclear translocation of p50 in primary human fibroblasts

Activation of the NFkB transcription factors are one of the early steps during viral infections, the activation of the pathways involving the homo and hetero-dimmers of p50 have implications on induction of a pro-inflammatory set of genes and in the regulation of the inflammatory response [38]. To evaluate if the infection with DENV induces transcriptional activation of the pro-inflammatory transcription factor, we evaluated the localization of p50 by IF in infected cells. At 48 h post-infection, p50 was observed in the nucleus of both infected and in neighbor cells with no evident infection, suggesting once again a paracrine effect as consequence of the antiviral/pro inflammatory micro environment by the infected fibroblasts. Figure 6F.

### DENV-2 clinical isolate induce apoptosis in human skin fibroblasts

**Figure 7:**
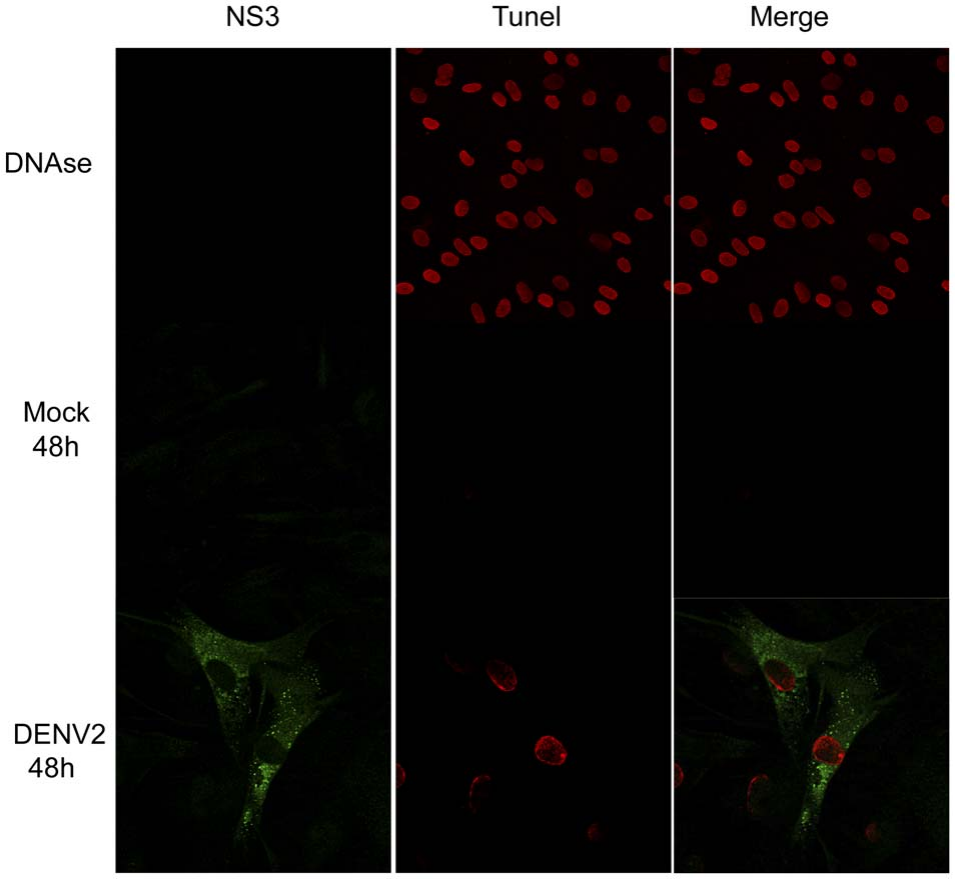
DENV-2 infection induced DNA fragmentation in skin primary fibroblasts. Skin fibroblasts were infected with 5 MOIs of DENV-2 or mock infected, and then fixed and stained with mouse anti-NS3 protein (green) followed by FITC-conjugate anti-mouse and then TUNEL (red) reaction was performed. Apoptosis of DENV-infected skin fibroblasts cells were evaluated at 24 and 48 hr post-infection by epifluorescence microscopy. Representative staininings of infected and mock-infected cells at 24 hours post- infection.

Some viruses direct the apoptosis of infected cells. The induction of apoptosis may be a viral strategy to aid dissemination. Furthermore, apoptosis has recently been associated with the activation of innate immunity pathways in DENV disease via TRADD caspase activation [39]. To evaluate whether Dengue virus serotype 2 is able to induce apoptosis in human primary fibroblast cultures, we infected primary skin fibroblasts with 5 MOI of DENV and then analyzed them at different times. Twenty-four hours after infection, 34% of the 60% of skin primary fibroblasts that expressed viral NS3 protein, showed a TUNEL-positive signal as detected by immunofluorescence (Figure 7). The maximum infected cell count (68% of the antigen-positive cells) was reached at 48 h after infection. Mock-infected cells do not shown viral antigen (NS3).

## Discussion

Cumulative invaluable data has been generated regarding the adaptive immune response against Dengue, focusing in characterizing the most important changes during the disease, such as those involving antibodies, T cells and cytokines [40], [41]. However, insufficient information is available regarding the early events of the innate immune response when the virus is introduced through the skin, the natural route to acquire the infection.

Given that mosquitoes inoculate DENV-2 into human skin while they are feeding, some research have suggested that DENV initial replication should occur in the skin and that potential target cells for dengue infection should be the cutaneous DCs localized in the epidermal and the dermal layers. [5], [9], [42] Recent evidences suggest that Langerhans and/or dendritic cells may take up antigens for processing and presenting them to the adaptive immune system, instead of working as reservoir cells for the dengue virus [43], [44].

Clearly other non-hematopoyetical cutaneous cells may potentially be infected; fibroblasts are one of the most abundant cell types at the Dengue virus arriving site. In consequence these cells may be physiologically relevant for in vivo infection when inoculation occurs by any virus-bearing tick or mosquito.

In this article we have shown that fresh human fibroblasts obtained from the healthy skin explants are permissive to DENV infection. These data were generated after double staining with markers for fibroblasts and DENV. This experimental model was used to more closely approximate an in vivo infection. Based on these data, we established human primary dermal fibroblasts cultures with a reduced number of passages, and we clearly demonstrated productive infection in all of primary cultures. Thus, healthy human skin fibroblasts are likely to be involved in the development of innate immune responses to arthropod or thick-borne viruses rather than being only target cells whose primary role has been proposed to serve as replication depot.

However it is important to consider that cumulative work raised using a wide range of models, with different hosts, mosquito species and arthropod-borne viruses showing that, mosquito saliva and/or feeding is associated with a potentiation or control, affecting virus transmission, host susceptibility, viremia and disease progression impairing the antiviral immune response [45], [46].

In contrast in other experimental data, the expression of anti-dengue effectors molecules in the distal-lateral lobes of *A. aegypti* salivary glands has shown to reduce prevalence and mean intensities of viral infection [47]. Moreover, Thangamani et al. demonstrated important differences in the activation of immune response to CHIKV due the route of transmission showing an up-regulation of TL3 in both uninfected and CHIKV infected mosquito bites [48]. This data showed contrasting immune activation in response to CHIKV. However other studies have shown no effect on arbovirus infection due to mosquito transmission

In summary the studies investigating the effects of mosquito saliva upon the vertebrate immune response remain unclear and all together may suggest that at high concentrations some salivary proteins are immmunosuppressive, whereas lower concentrations of this modulate the immune response [49].

Understanding the potential role of mosquito saliva over the DENV infected fibroblasts is a point that must be considered in the future.

Double-stranded RNA is produced during DENV viral replication. This molecule can be detected by Toll-like receptors (TLRs) 3 and 7with in the endosomes, while the RNA helicases detect viral dsRNA in the cytoplasm. It has been described the possibility that DENV is detected by RIG-I, TLR3 and TLR7 (depending on the cell type) triggering the IFN response [16], [18], [19], [21]–[23]. These detection systems are not mutually exclusive and the expression of the PRRs depends on the cell lineage or origin.

Our data showed that in the primary skin fibroblasts, both TLR3 and RIG-1 (but not Mda5) molecules are up-regulated at different times suggesting that both molecules may trigger a coordinate induction of the antiviral response against DENV. TLR3 may “prime” the early response while RIG-I might regulate the amplified response at later time points. This concerted response has been observed in a model of MEFs infected with WNV, in which RIG-I mediated the initial detection of WNV infection in cells of non-immune origin. But RIG-I-deficient cells were still capable of responding to WNV, suggesting that other PRRs are also involved in mediating the innate antiviral response.

During DENV detection by skin fibroblasts, none of the established cultures showed mRNA for TLR7 after DENV infection. These data may suggest the absence of these PRRs in such cells. Similarly, Paladino *et al.* analyzed the TLR expression in virally infected fibroblasts of different origins and found absence of TLR7 [10].

The differences in the molecules involved in DENV detection suggest a differential cytokine profile that may contribute and maintain the microenvironment collaborating with dendritic cells (DCs) and possibly other resident cells when the virus arrives. The activation of the resident DCs depends on viral replication itself and also on the presence of important mediators such as IFNβ, TNFα and defensins, and they may be modulated by activation of other signaling pathways, such as the production of double-stranded RNA.

During viral infection various cytokines play a role both in viral clearance and in tissue damage mechanisms. The type I interferon's (IFNs) is regulated by a large family of multifunctional immunoregulatory proteins such as IRF3 and IRF7. IRF3 possesses a restricted DNA binding site specificity and interacts with CBP, while IRF-7 has a broader DNA binding specificity that contributes to its capacity to stimulate IFNα subtype expression. Both IRF3 and IRF7 play distinct and essential roles in the IFNα/β response to eliminate the viral infection. At the same time the activation of transcription factors during the infection depends on the signal given to the PRRs. Here we observed activation of IRF3, and we also observed that even if the secretion of IFNβ maintains the temporal kinetics, the magnitude of the response varies between donors. Moreover, studying skin samples from different donors, races and ages, would give valuable information of the innate immune system profile that may correlate with the susceptibilities of some hosts.

On other hand if the skin fibroblasts produces type I interferons this event becomes very important in terms of contribution to an antiviral state, in the infection site, more when considering that DENV is a strong inducer of type I IFN responses.

The production of defensins by the infected fibroblasts may increase the recruitment of immune cells such as neutrophils and monocytes during the DEN infection; otherwise these molecules may act directly causing virolisis.

Preliminary experiments in our laboratory evaluating the potential activity of human defensins over the viral particle suggests a direct activity against DENV (unpublished data).

RIG-I activation induces the association with IPS-1 (Cardiff, Visa or MAVS). This interaction induces the activation of the inflammatory/antiviral response. However, the exposure of the Caspase recruitment domains (CARD) of RIG-I and IPS-1 may also recruit TRADD, RIP-1 and FADD, a molecular complex known as the TRADDosome, which induces the activation of caspases 8 and 10 provoking cell death [39]. We detected cell death early during the infection with DENV (Figure 7) and an increment of the RIG-I protein (Figure 3D). The apoptosis activation may be caused by the activation of the innate immune system; however, more extensive studies are needed to test this hypothesis. These data corroborate findings previous findings by our group [50].

Clearly most studies addressing innate immunity in Dengue have been performed using human or mouse cell lines; however, not enough work has been performed by using primary human cultures of cells from the natural host and from the first anatomical site in contact with DENV when it enters through the natural route. These cells produce soluble mediators such as IFNs, TNFα and defensins. The magnitude and the variety of this response will depend on the amount of virus load inoculated and in consequence it might limit the infection *in situ*. Some studies suggest that the localization of the fibroblasts may confer different phenotype and gene expression. [51], [52] However we used healthy skin obtained from arm, abdomen and leg among others, and no differences in terms of permissively to DENV infection were detected.

Whether the mediators produced by dermal fibroblasts contribute mainly to pro-inflammatory anti-viral responses, or whether these cells might be used as in vivo reservoirs where an initial DENV replication occurs, is still uncertain and deserves further scrutiny.

As far as we know this is the first study where primary skin fibroblast cultures from different individuals have been evaluated for their susceptibility to DENV infection and for the elements of innate immunity that may contribute to DENV antiviral state. Moreover, this is the first report showing that defensins may be up-regulated by skin cells in response to DENV infection.

## Supporting Information

Figure S1 **Establishment and characterization of skin fibroblast cultures.** A) Details from ten donors with the corresponding percentages of infection. B) Once the cultures where established they were characterized with an antibody against fibroblast protein (IB10) by flow cytometry to assess the homogeneity of the culture. C) Expression of E glycoprotein in the skin donors detected by cytometry by using an anti- E monoclonal antibody.
(TIF)
