# Supplementary figures and images for: Activation of the Innate Immune Response against DENV in Normal Non-Transformed Human Fibroblasts
Source: PLoS Negl Trop Dis. 2011 Dec 20;5(12):e1420. doi: 10.1371/journal.pntd.0001420 (PMC3243703; doi:10.1371/journal.pntd.0001420)

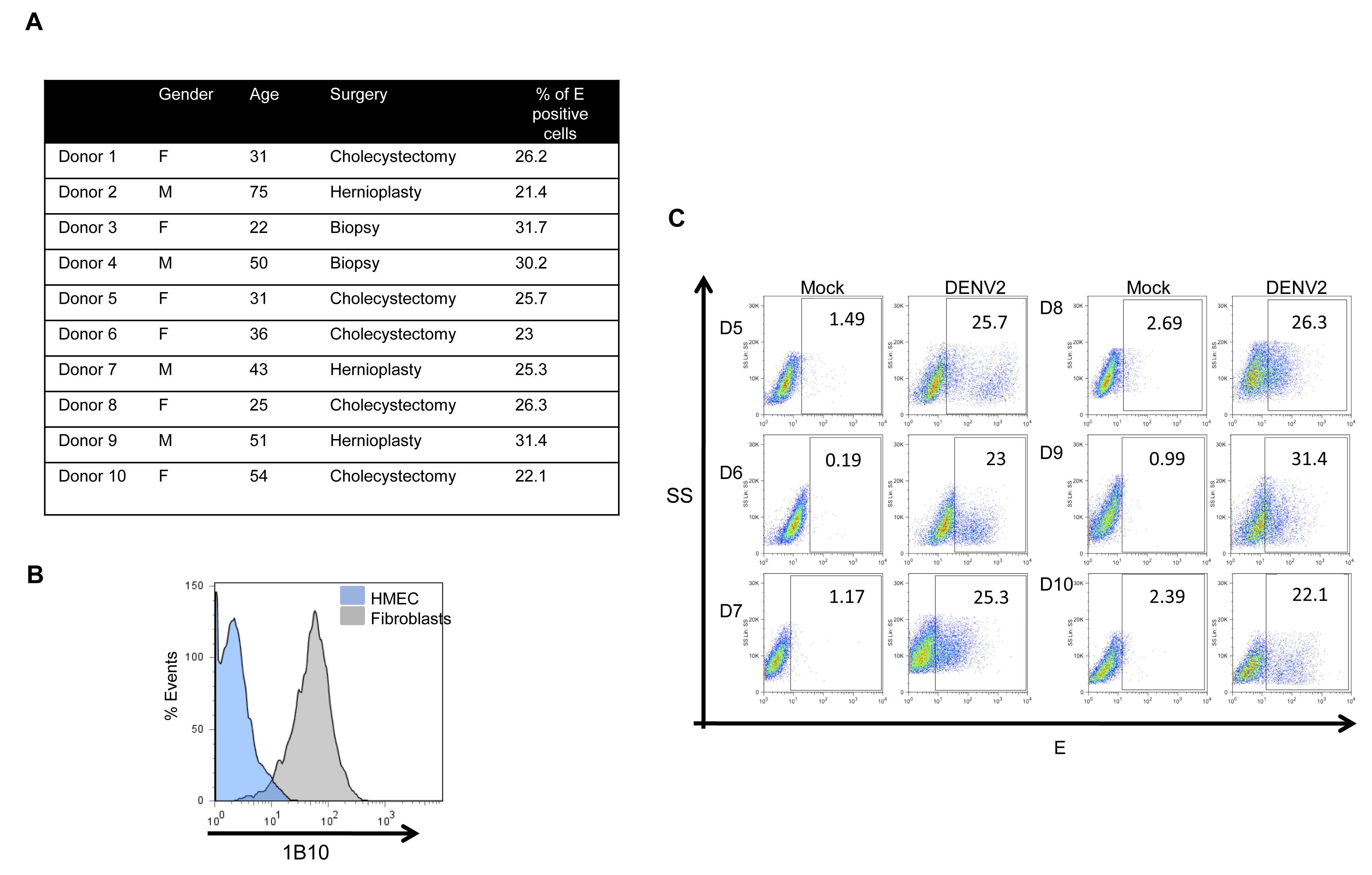

Supplement: Figure S1 — Establishment and characterization of skin fibroblast cultures. A) Details from ten donors with the corresponding percentages of infection. B) Once the cultures where established they were characterized with an antibody against fibroblast protein (IB10) by flow cytometry to assess the homogeneity of the culture. C) Expression of E glycoprotein in the skin donors detected by cytometry by using an anti- E monoclonal antibody. (TIF) [file pntd.0001420.s001.tif]
